# Improvement of Accuracy in Environmental Dosimetry by TLD Cards Using Three-dimensional Calibration Method

**Published:** 2015-06-01

**Authors:** S. J. HosseiniAliabadi, S. M. Hosseini Pooya, H. Afarideh, F. Mianji

**Affiliations:** 1 Nuclear Safety & Radiological Protection Research Department, Nuclear Science & Technology Research Institute, AEOI, Tehran, Iran; 2 Faculty of Energy Engineering & Physics, Amirkabir University of Technology, Tehran, Iran

**Keywords:** Accuracy, Calibration, Dosimetry, Environmental, TLD

## Abstract

**Introduction:**

The angular dependency of response for TLD cards may cause deviation from its true value on the results of environmental dosimetry, since TLDs may be exposed to radiation at different angles of incidence from the surrounding area.

**Objective:**

A 3D setting of TLD cards has been calibrated isotropically in a standard radiation field to evaluate the improvement of the accuracy of measurement for environmental dosimetry.

**Method:**

Three personal TLD cards were rectangularly placed in a cylindrical holder, and calibrated using 1D and 3D calibration methods. Then, the dosimeter has been used simultaneously with a reference instrument in a real radiation field measuring the accumulated dose within a time interval.

**Result:**

The results show that the accuracy of measurement has been improved by 6.5% using 3D calibration factor in comparison with that of normal 1D calibration method.

**Conclusion:**

This system can be utilized in large scale environmental monitoring with a higher accuracy.

## Introduction

TLD cards are widely used as personal dosimeters for individual monitoring. However, it can be used for environmental monitoring due to its special characteristics in dosimetry [1].

The natural external radiation dose to the public varies in different parts of the world based largely on the geomagnetic field, altitude and solar cycle. External dose from terrestrial radiation varies largely with variations of concentrations of radioactive potassium and the radioactive components of the natural uranium and thorium radioactive decay chains. Stochastic effects such as cancer and genetic disorders may be caused when living creatures are exposed to low doses. So the accurate measurement technique is an important subject in environmental dosimetry.

Most of commercial TLD readers have been designed based upon TLD cards which are used for individual dosimetry. These cards are inclusive of TLD pellets or chips which have angular dependency of response in radiation fields [2, 3]. This angular dependency should be calculated for personal dosimetry uncertainties and compared against the trumpet curve of ICRP criteria [4]. But the uncertainty cannot be developed for environmental monitoring in which low dose values and high accuracy measurements are the main objectives.

In this research, a new setting of TLD cards has been developed for environmental dosimetry. The dosimeters have been calibrated isotropically in a standard radiation field. Then, the response of the dosimeter has been compared with a reference instrument in a real radiation field. 

## Materials and Methods

**Figure 1:**
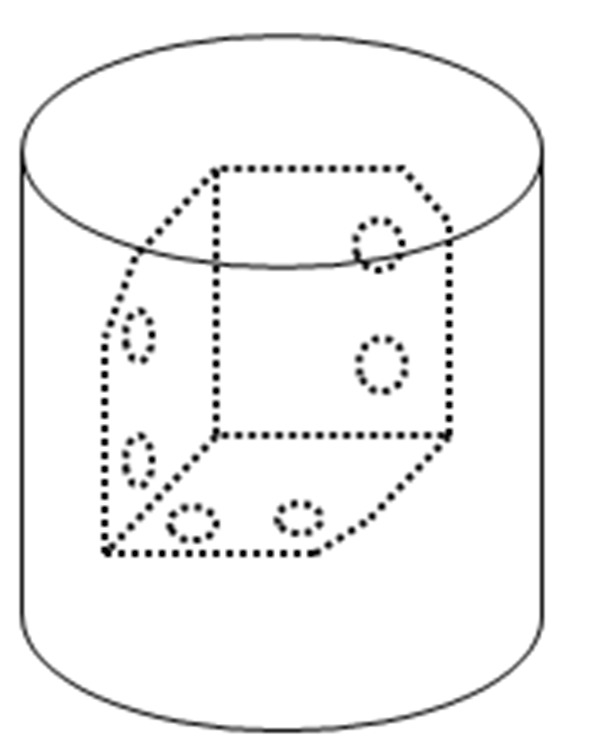
Schematic of a 3D TL Dosimeter in a Cylindrical Al Wall for Environmental Measurements

Figure 1 shows the schematic views of three personal TLD cards (TLD-100) which are rectangularly placed in a cylindrical aluminum holder wall with thickness of 2 mm to minimize the penetration of β-ray from the ambient. Each TLD card was placed between two Plexiglas layers with thickness of 2 mm to satisfy CPE conditions. All three dosimeters have been calibrated using standard calibration method at 4 different ambient doses equivalent values by a^137^Cs source field which is traceable with a secondary standard dosimetry Laboratory.

**Figure 2:**
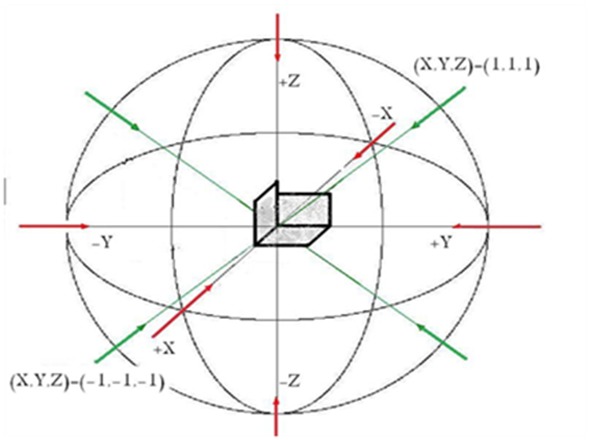
Schematic of a Three-dimensional Calibration Method with Ten Angles of Incidence

Figure 2 shows the three-dimensional calibration exposures at ten selected angles coinciding with vectors; (1,0,0), (-1,0,0), (0,1,0), (0,-1,0), (0,0,1), (0,0,-1), (1,1,1), (-1,-1,-1), (-1,1,1), (1,-1,-1).

**Figure 3:**
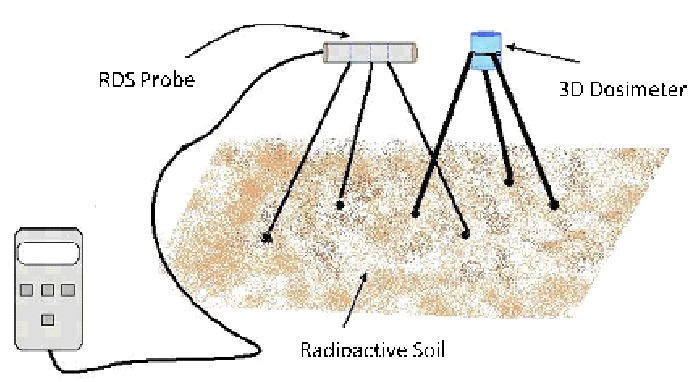
Configuration of an Experimental Test of the 3D Dosimeter Response in a Real Radiation Field

In order to evaluate the accuracy of the three-dimensional calibration method, the 3D dosimeter system was placed at the height of 1 meter above a wide stack of homogenous radioactive soil (figure 3). The radioactive soil has been provided from the high-level natural radiation area (HLNRA) of Ramsar containing high concentration of ^226^Ra and its decay products[5].

A calibrated cylindrical probe of a survey-meter RDS-110 model was used as the reference instrument at the same height from the source near the 3D dosimeter. The windowless probe was placed in parallel with the ground state direction. The RDS-110 detects gamma and X-rays with energies from 50 keV up to 1.3 MeV. This device can measure the radiation dose in the range of 0.001–999.9 mSv, and the dose rate from 0.05 mSv h^–1^ to 99.99 mSv h^–1^ [6].

## Results and Discussion

Calibration factors of the TLD cards in 1D and 3D calibration methods have been presented in table 1. As it can be seen in the table, the calibration factors in 3D method are greater than that of 1D method. Because of the angular dependency of the cards, it is predictable that the smaller TL values, the greater calibration factors.

**Table 1:** Results of Calibration Factors of TLD Cards in 1D and 3D Calibration Methods

Calibration Method	Card ID #1	Card ID #2	Card ID #3
1D Calibration Factor of Cards	0.119	0.117	0.116
3D Calibration Factor of Cards	0.130	0.125	0.123
3D Calibration Factor of the 3D Dosimeter System	0.126		

On the other hand, the reference instrument showed a value of 3.84 ± 0.13 mSv of accumulated ambient dose equivalent in the experiment setting of Figure 3. The related accumulated doses which were measured by TLD cards using different calibration methods have been presented in table 2. The absolute deviations in 1D and 3D calibration methods have been obtained 11.7% and 5.2%, respectively i.e. the 3D calibration method improves the accuracy of measurements by 6.5% in value.

**Table 2:** The Results of Absolute Deviations of TLD Responses Using 1D and 3D Calibration Factors

**Card ID**	**Dose value in 1D cal. (mSv)**	**Absolute deviation from reference value (mSv)**	**Percentage of the average of deviations in 1D cal.**	**Dose value in 3D cal. (mSv)**	**Absolute deviation from reference value (mSv)**	**Percentage of the average of deviations in 3D cal.**
#1	3.41	0.43	11.7%	3.64	0.20	5.2%
#2	3.13	0.71				
#3	3.64	0.20				

This improvement in accuracy strongly depends upon the precision in calibration method. In other words, the greater the number of selected points of dose values in calibration method, the better improvement of accuracy in 3D calibration method. 

## Conclusion

A new setting of TLD cards has been introduced to achieve a more accurate environmental dosimetry. The system has been calibrated isotropically, and tested in a real radiation field.  

The results show that the accuracy of measurement has been improved using three-dimensional calibration factor in comparison with that of normal calibration method. So, the system can be used in large scale environmental monitoring such as national and regional gamma monitoring programs with higher accuracy. 
